# Fungal feelings in the irritable bowel syndrome: the intestinal mycobiome and abdominal pain

**DOI:** 10.1080/19490976.2023.2168992

**Published:** 2023-02-01

**Authors:** Iam van Thiel, Wj de Jonge, Rm van den Wijngaard

**Affiliations:** a Tytgat Institute for Liver and Intestinal Research, Amsterdam University Medical Centers, Amsterdam, The Netherlands; b Amsterdam UMC, University of Amsterdam, Gastroenterology and Hepatology, Amsterdam Gastroenterology Endocrinology Metabolism, Amsterdam, The Netherlands; c Department of Gastroenterology and Hepatology, Amsterdam University Medical Centers, Amsterdam, The Netherlands; d Department of General, Visceral-, Thoracic and Vascular Surgery, University Hospital Bonn, Bonn, Germany

**Keywords:** *Candida albicans*, IBS, irritable bowel syndrome, macrophage, mast cells, microbiome, mycobiota, therapy, visceral hypersensitivity, yeast

## Abstract

Although the gut microbiota consists of bacteria, viruses, and fungi, most publications addressing the microbiota-gut-brain axis in irritable bowel syndrome (IBS) have a sole focus on bacteria. This may relate to the relatively low presence of fungi and viruses as compared to bacteria. Yet, in the field of inflammatory bowel disease research, the publication of several papers addressing the role of the intestinal mycobiome now suggested that these low numbers do not necessarily translate to irrelevance. In this review, we discuss the available clinical and preclinical IBS mycobiome data, and speculate how these recent findings may relate to earlier observations in IBS. By surveying literature from the broader mycobiome research field, we identified questions open to future IBS-oriented investigations.

## Introduction

Irritable bowel syndrome (IBS) is a common disorder of gut-brain interaction that affects an approximate 5–10% of the population. Diagnosis is based on symptoms and exclusion of structural alterations. Patients experience chronic abdominal pain in combination with altered bowel habits.^1^ Online searches suggest that at least part of the IBS patients and clinical practitioners is convinced that intestinal yeasts are causally involved in this disorder (Google Search; “irritable bowel syndrome AND yeast”; 4,410,000 results, January 2023). Yet, scientific evidence for such an assumption is scarce. In PubMed, a search string including “irritable bowel syndrome AND (yeast OR candida OR myco*)” showed 152 results (January 2023). Although IBS-oriented gut microbiome research exploded during the last decade, investigations were mostly geared toward bacteria, leaving fungi (including yeasts) largely unexplored.^2,3^ The latter may be explained by the finding that fungi are only a minor component of the gut ecosystem. Shotgun sequencing results published by the METAgenomics of the Human Intestinal Tract (MetaHIT)-consortium indicated that fungi constitute no more than 0.1% of the human gut microbiota,^4^ although this may have been an underestimate due to the use of non-yeast directed DNA isolation procedures and deficient fungal genome databases used for annotation. Another important consideration is that fungi are approximately 100 times larger in size than bacteria. Relative biomass is not reflected in read counts of metagenomics approaches but may be important when considering the fungal contribution to the total gut metabolome. Compiling 36 human gut mycobiome studies, Suhr and Hallen-Adams^5^ observed that ten out of the twelve most commonly detected fungi are yeasts, with *Candida albicans* and *Saccharomyces cerevisiae* as leading species. This may be relevant because yeast are capable of fermentation, and a diet low in fermentable oligosaccharides, disaccharides, monosaccharides, and polyols (FODMAPs) was shown to manage IBS symptoms in the short and long term.^6^ Importantly, whereas *C. albicans* is considered a gut commensal, the ubiquitous dietary presence of *S. cerevisiae* may explain at least part of its high frequency of detection.^7^ In the field of inflammatory bowel disease (IBD), there is an increasing awareness that intestinal fungi/yeasts may be relevant players in IBD pathogenesis.^8^ In IBS, mycobiome research is still in its infancy, despite patients’ perception and attitude toward the subject. Yet, some small IBS cohort studies indicated alterations of the fecal fungal community with, amongst others, enhanced relative abundance of *C. albicans*. In addition, using the IBS-like maternal separation model in rat, the first preclinical studies on the functional relevance of the mycobiome and modulation thereof have been published.^9–15^ In this review, we aim to discuss the sparse IBS mycobiome literature and some of the recent developments in the broader IBS- and mycobiome research fields that may be relevant in the IBS/mycobiome context as well.

## Anti-fungal and anti-food allergen antibodies

**Figure 1.**
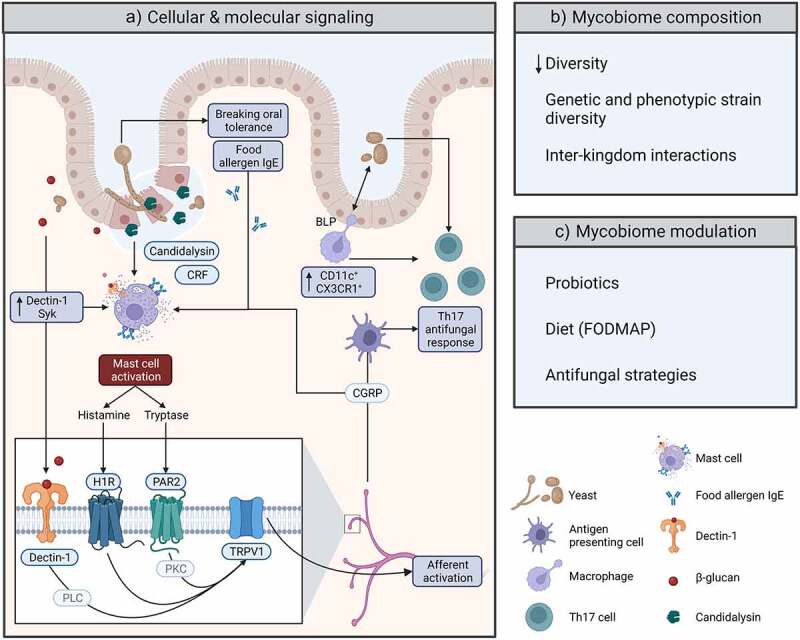
Possible mycobiome-related mechanisms of action in Irritable Bowel Syndrome. (a) Cellular and molecular mechanisms through which host and mycobiome may interact. Part of these interactions were described in IBS and IBS-like rodent models. Others were addressed in the somatic pain field and still need confirmation in IBS (see text). BLP, balloon-like protrusion; CGRP, calcitonin-related gene peptide; CRF, corticotropin-releasing factor; H1R, histamine-1-receptor; PAR2, protease activated receptor 2; PKC, protein kinase C; PLC, phospholipase C; TRPV1, transient receptor potential channel vanilloid 1. (b) Recent observations and considerations regarding the gut mycobiome in IBS. (c) Possible strategies for modulation of the fungal community in patients with IBS. Illustration created with BioRender.com.

One of the early findings that sparked mycobiome research in IBD was the serological presence of the so-called anti-*Saccharomyces cerevisiae* antibodies (ASCAs), especially in patients with Crohn’s disease (~60%).^16^ These antibodies recognize yeast mannans and we now know that *Candida albicans* is the more likely fungal immunological antigen trigger for ASCAs.^17^ Serum titers in IBS were addressed in several studies, but ASCAs never showed increased presence over healthy volunteer sera.^18–20^ Although this may indicate that there is no role for intestinal fungi, it could also relate to clear differences in (immune)-pathology between IBD and IBS. The latter is characterized by a general absence of overt inflammation,^21^ whereas in Crohn’s disease, ASCA titers were shown to correlate with disease severity.^22,23^ Even so, antibodies with other specificity, i.e., dietary antigens, have been implicated in mast cell-dependent visceral pain. Mast cells and especially the mast cell mediator histamine are well-recognized drivers of abdominal pain complaints in at least a subset of IBS patients.^24–26^ In a study conducted by the Boeckxstaens group, mucosal mast cells of all IBS patients proved to be sensitive to at least one of the tested food allergens upon local injection into rectosigmoid mucosa, despite negative skin prick tests.^24^ The investigators suggested that bacterial gut infections and subsequent epithelial barrier disruption are the initial trigger for this localized immune response (Figure 1a). The latter was modeled in the ovalbumin (OVA) food allergy mouse model. While exposing mice to OVA, they were simultaneously infected with *Citrobacter rodentium*. When the infection was cleared, repeated OVA exposure led to an enhanced IgE-specific and mast cell-dependent visceromotor response during colorectal distension that was not observed in sham infected mice. The visceromotor response is often used in preclinical studies as readout for visceral sensitivity.^27,28^ Visceral hypersensitivity (i.e., increased perception of gastrointestinal stimuli) was shown to consistently associate with symptom severity in functional gastrointestinal disorders and is thought to be a mechanism explaining abdominal pain.^29^ Based on the OVA/*C. rodentium* results, it was suggested that transient gut infection, like it is observed in post-infectious IBS (PI-IBS), can lead to IgE-dependent food allergy and abdominal pain.^24,30^ Others have shown that *C. albicans* is also capable of breaking oral tolerance in the OVA model and confirmed a role for IgE and mast cells, but possible effects on visceral sensitivity were not addressed in these *C. albicans*-focused studies.^31,32^

## Mast cells and visceral hypersensitivity

Possible triggers for mast cell activation include, next to the aforementioned (food allergen-specific) IgE, a broad range of nonimmune signals such as substance P, nerve growth factor, calcitonin gene-related peptide (CGRP).^33^ Because stress is an important trigger for IBS-associated complaints, the stress hormone corticotropin releasing factor (CRF) received a lot of attention. Stress-induced mast cell-dependent visceral hypersensitivity is thought to be an important mechanism explaining abdominal pain in IBS. Preclinical IBS-like animal models suggested that peripheral CRF can be responsible for mucosal mast cell degranulation, subsequent gut barrier dysfunction, and visceral hypersensitivity (Figure 1a).^33,34^ Yet, clinical trials with CRF-receptor antagonists were unsuccessful,^35,36^ possibly due to inadequate antagonist development.^37^ In search for an alternative explanation, we addressed a possible flaw in the design of pioneering preclinical IBS studies that mostly administered CRF-receptor antagonists prior to stress.^34^ When maternal separated rats were subjected to acute stress at adult age (i.e., 1 hour of water avoidance stress), they became hypersensitive to colorectal distension. This visceral hypersensitivity developed directly after water avoidance and was mediated by the activation of mucosal mast cells.^28,38^ We next showed that visceral hypersensitivity remained to be present for up to 30 days after just 1 hour of water avoidance stress, and that this long-term hypersensitivity also depended on the activation of mast cells.^39^ Administering the CRF-receptor antagonist α-helical CRF prior to water avoidance stress prevented visceral hypersensitivity, but post-stress administration of the same antagonist could not reverse long-term hypersensitivity. In other words, acute and long-term visceral hypersensitivity both depended on mast cells, but only in the acute phase their activation was triggered by CRF. In a follow-up investigation, described in the next paragraph, we used the maternal separation model to address the possible role of intestinal fungi in post-stress mast cell activation and prolonged visceral hypersensitivity.^9^

## The mycobiome and visceral hypersensitivity

To assess whether the gut mycobiome is involved in IBS, we first compared the fecal mycobiota of healthy volunteers and patients with IBS with known visceral sensitivity status. Patient samples showed lower α-diversity and we observed differences in mycobiome signature when comparing normosensitive and hypersensitive patients.^9^ Importantly, mycobiota dissimilarities between controls and IBS patients were later confirmed by others.^12–14^ Subsequent analysis of fecal samples obtained from normal and maternal separated IBS-like rats showed profound mycobiome differences. In this mast cell and histamine-1-receptor dependent model, fungicide treatment reversed stress-induced visceral hypersensitivity.^9,38,40^ Additional fecal transfer experiments confirmed an essential role for the maternal separation-related gut mycobiome. In relation to these results, it should be re-emphasized that despite the obvious role of intestinal fungi and mast cells, there is no overt inflammation in this IBS-like model.^38^ The latter properly reflects the immune status in IBS.^21^ Interestingly, although *C. albicans* is not considered a common colonizer of the rodent gut,^41^ our rat mycobiome analysis indicated fecal presence of this species. This may be due to non-sterile handling procedures and the open cage system that was used for the Long-Evans in-house rat colony at the time. Although this may complicate duplication studies by other labs, we consider the use of “dirty rats” a positive attribute. Laboratory rodents are generally kept in highly sanitized environments, avoiding any contact with novel (pathogenic) microbial components. This does not reflect the everyday situation in humans and may diminish the translational value of these models.^8^ Yeung *et al*. showed that controlled release of lab mice into the wild led to increased intestinal fungal load and, related to that, immune system maturation. The latter showed in enhanced differentiation of T cell populations and increased numbers of circulating granulocytes.^42^ Although this “rewilding” setting is different from what our rats experienced, we assume that the translational value of our model was positively affected by the suboptimal level of cleanness in which experiments were conducted. Yet, we did not address whether the presence of *C. albicans* in maternal separated rats was essential for the hypersensitive phenotype. In IBS patients however, *C. albicans* was shown to be more abundant and the genus *Candida* positively correlated to severity of bloating and anxiety in diarrhea-predominant IBS (IBS-D).^12,13^ It has to be noted though that these sequencing results were obtained based on fecal samples. While these samples are relatively easy to obtain, they may not provide an accurate window to colonic mycobiota composition at mucosal level. Compared to healthy volunteer samples, the relative abundance of fecal *Candida* spp. is consistently increased in IBD cohorts, and recent data obtained by Li *et al*. showed that this is mirrored in ulcerative colitis mucosa.^43^ Since there are no mucosa-associated mycobiota studies in IBS, it is not known whether the same holds true for this disorder.

## Mast cells and the mycobiome in visceral hypersensitivity

Prior to our mycobiome-directed investigations, several groups already described direct fungal-induced mast cell activation involving the C-type lectin receptor Dectin-1 (Figure 1a).^44–46^ This transmembrane receptor is predominantly expressed by myeloid cells and recognizes 1,3-linked β-glucans present in the fungal cell wall. Spleen tyrosine kinase (SYK) and caspase-associated recruitment domain 9 (CARD9) are both essential in mediating downstream cellular responses of this receptor.^47^ Given the relevance of mast cells in sensory afferent activation in patients as well as in the maternal separation model,^24–26,38–40^ we also addressed this particular pathway of innate fungal recognition. Administration of inhibitory soluble β-glucans that antagonize Dectin-1, as well as a SYK inhibitor both reduced visceral hypersensitivity in maternal separated rats. *Ex vivo* experiments with mesenteric windows confirmed Dectin-1 dependent mast cell degranulation by particulate β-glucans.^9^ In human, indications for a role of the Dectin-1/Syk pathway in mast cell activation were obtained by Chi *et al*.^48^ When comparing IBS-D and healthy volunteer ileocecal biopsies, these investigators observed enhanced expression of *CLEC7A* (encoding Dectin-1), *SYK*, and *CARD9* in patient biopsies. Which cell types were responsible for the enhanced expressions cannot be deduced from their findings. However, there was a positive correlation between plasma tryptase levels and *CLEC7A* expression, as well as plasma tryptase and increased visceral sensitivity of these patients. Next to mast cell derived histamine that triggers visceral hypersensitivity via the histamine-1-receptor in the maternal separation model and patients, tryptase was implicated in IBS pathophysiology by others as well.^26,33,40^ This serine protease is released during mast cell degranulation. It can activate proteinase-activated receptor 2 (PAR2) on sensory afferents and enterocytes, leading to visceral hypersensitivity and barrier dysfunction (Figure 1a).^49–51^ Concerning its mechanisms of action, cell culture experiments showed that activated PAR2 sensitizes, via protein kinase C, the capsaicin receptor transient receptor potential channel vanilloid 1 (TRPV1). Moreover, in an *in vivo* somatic pain setting, co-administration of non-hyperalgesic dosis of PAR2 agonist and capsaicin led to hyperalgesia that was absent in *Trpv1*^−/−^ mice.^52^ Since TRPV1 is also a key ion channel for visceral hypersensitivity in maternal separated rats and patients with IBS,^26,38^ these somatic findings on PAR2-mediated afferent sensitization may be relevant for IBS as well. Indeed, results obtained in an acetic enema rat model of visceral hypersensitivity suggested that the mast cell-PAR2-TRPV1 axis may play a role.^53^ The possible relevance of tryptase/PAR2-induced intestinal permeability becomes clear knowing that barrier dysfunction observed in patients with IBS-D and PI-IBS subtypes positively associates with abdominal pain and changes in bowel function.^54^ Moreover, when stress-induced barrier dysfunction was prevented in a rat model for IBS, this also prevented the development of visceral hypersensitivity.^55^ Taken together, the above data suggest that mast cell recognition of intestinal fungi, via the pattern recognition receptor Dectin-1, may lead to histamine and tryptase release and consequent barrier dysfunction and visceral hypersensitivity.

### Candida albicans *and candidalysin dependent afferent activation*

Dectin-1 dependent abdominal pain may also result from direct afferent activation. Albeit in a somatic setting, it was shown that *C. albicans* can interact with afferent expressed Dectin-1 to activate the neuronal phospholipase C/TRP-channel axis (i.e., TRPV1 and TRPA1; Figure 1a) and cause β-glucan-dependent allodynia.^56^ Importantly, in addition to showing a role for direct afferent activation in fungal-induced somatic pain, the same study also suggested that keratinocytes were the main cell type responsible for Dectin-1 dependent allodynia. In response to *C. albicans*, these skin epithelial cells released ATP that subsequently stimulated sensory afferents via P2X receptors. Whether similar mechanisms are relevant for visceral pain is not known, but functional Dectin-1 may be absent from enterocytes.^57^ Besides addressing a role for Dectin-1, Maruyama and colleagues also investigated a possible role for candidalysin in direct afferent activation.^56^ This pore-forming peptide was the first cytolytic toxin identified in *C. albicans*. Although *C. albicans* is a gut commensal, its ability to transform from yeast to filamentous morphology can turn it into an opportunistic pathogen. For in depth coverage and excellent graphical visualization of *C. albicans* interaction with, and translocation across the intestinal barrier and discussion of all virulence factors involved in this process, please refer to a recent open access publication by Sprague *et al*. in this journal.^58^

Concerning candidalysin, it was shown that hypha-associated candidalysin is an essential virulence factor leading to damage of host cells and concurrent activation of anti-fungal immune responses.^59,60^ Others had shown that the bacterial pore-forming toxin α-hemolysin induces calcium flux and action potentials in nociceptor neurons.^61^ Candidalysin however did not induce calcium fluxes in dorsal root ganglion neurons isolated from mice.^56^ Mast cells on the other hand may be stimulated by candidalysin (Figure 1a). Using HMC-1 and LAD2 mast cell lines, candidalysin peptide and candidalysin-positive *C. albicans* strains were both shown to induce degranulation and cytokine release, which was not observed when candidalysin-null mutant *C. albicans* strains were used. Furthermore, intradermal (i.e., skin) injection of candidalysin in wild-type mice led to vascular permeability that was not observed in mast cell deficient KitW-sh/W-sh mice.^62^ At present there are no data regarding *in vivo* candidalysin-mediated degranulation of colonic mast cells. But, some other candidalysin-focused investigations, described in the section on “*C. albicans* strain differences”, were performed in relation to IBS.^15^

### Candida albicans *morphotype switching and IBS*

As mentioned earlier, candidalysin becomes expressed upon yeast to hyphae transition which can be triggered by a diversity of signals such as temperature, pH, serum, N-acetylglucoseamine, peptidoglycan, amino acids *etcetera*. Because of the multiplicity and complexity of possible triggers and pathways involved in morphotype switching, Noble *et al*. suggested that *C. albicans* “continuously surveils the mammalian host, integrating a variety of signaling inputs to generate adaptive responses to the local environment”.^63^ For IBS, the nature of these local environmental triggers is unknown. Even more, at this point there are no publications regarding the presence or absence of filamentous cells in IBS fecal and/or mucosal samples. However, in a mouse model of *C. albicans* colonization, yeast and hyphal morphotypes were simultaneously present, herewith challenging the dogma that these appearances strictly belong to commensal and tissue-invasive states respectively.^64,65^ In the absence of tissue invasion, both forms were observed in the intestinal lumen as well as adjacent to and in the mucus layer. Compared to stomach and small intestine, the filamentous form even predominated over yeast in cecum and colon. Further investigations showed that not yeast-to-hypha transition per se (i.e. cell morphology), but activation of virulence factors controls *C. albicans* intestinal commensalism. Based on their observations, Witchley *et al*. suggested that an anti-fungal host response to the hyphal phenotype will only be triggered when virulence factors exceed a threshold level.^65^ This interesting “under the radar” hypothesis may also be relevant for IBS and the IBS-like maternal separation model, were fungi seem to play a role in abdominal pain without triggering a massive immune response.^9,66–68^

### Candida albicans *strain differences*

Pilot investigations in a limited set of fecal healthy volunteer and IBS patient-derived *C. albicans* strains showed that yeast-to-hyphae transition could be induced in all strains, whereas simultaneous *ECE1* (the gene encoding candidalysin) induction rates greatly differed.^15^ The IBS *C. albicans* strains were isolated from hyper- and normosensitive patients. Genotyping by Amplified Fragment Length Polymorphisms (AFLP)-fingerprinting analysis of 63 strains showed partial clustering of strains derived from hypersensitive patients. Unfortunately, *ECE1* expression analysis was performed in six strains only, thus preventing any conclusions regarding possible correlations with visceral hypersensitivity. On the other hand, clonal expansion of IBS-derived *C. albicans* strains distinct from healthy volunteers was also described by others, although without including the hyper- *vs*. normosensitive distinction.^14^ Notably, the functional relevance of *C. albicans* strain differences for IBS pathophysiology and IBS clinical characteristics was not addressed so far. In IBD however, a range of *C. albicans* clinical isolates showed different damaging capacity toward macrophages, and induced different levels of antifungal interleukin (IL)-17A-producing T helper cell (Th17) responses. *In vitro* evaluation of *C. albicans Ece1* knockout in high and low damaging strains suggested that candidalysin has a key role in these processes, and this was confirmed *in vivo*.^43^ Candidalysin is also critical to induce epithelial damage needed for *C. albicans* translocation across intestinal epithelia.^69,70^ As mentioned before, our first fungal findings in the maternal separation model suggested that β-glucans are an important trigger for mast cell activation and subsequent histamine-induced visceral hypersensitivity.^9^ In a review on host-microbe interactions, the question was raised how these fungal antigens make their way from the gut lumen.^71^ Knowing that *C. albicans* was present during the referred animal experiments, we now suggest that future investigations should focus on a possible role for candidalysin in yeast or its antigen translocation in IBS.

Increased attention for strain differences between healthy volunteers and patients with IBS may also accelerate the search for biomarkers in this disorder. Due to the lack of convincing biological indicators, IBS is diagnosed by the symptom-based Rome IV criteria.^72^ In search for alternative tools to discriminate patients with IBS from healthy individuals, or even identify patient subsets, numerous bacteria-focused microbiota analysis have been conducted on healthy volunteer and IBS fecal samples. Until now, inter study discrepancies prevented the identification of a universal “IBS microbiota” and hampered broad application of such techniques.^73^ Recent findings within the Dutch Microbiome Project suggest that this type of biomarker-finding efforts may even be more difficult than appreciated thus far. Bacterial microbiome profiling of more than 8000 Dutch individuals showed shared dysbiosis between unrelated diseases (including IBS), which will most likely complicate the identification of disease specific microbiome signatures.^74^ Similar inter-disease datasets are not yet available for the intestinal mycobiome, but its low abundance and high inter-individual variability already suggest that classical compositional analysis will most likely not result in satisfactory mycobiome based diagnostic and monitoring tools.^75^ In general, a major caveat in bacterial as well as fungal compositional microbiota analysis for biomarker finding, is the complete disregard of strain differences within a species. Odds *et al*. studied a large set of non-IBS-related *C. albicans* strains that included isolates from longitudinal samples. Their results suggested clonal persistence in time.^76^ Based on this, and the two recent publications on genetic clustering of *C. albicans* strains in IBS,^14,15^ we argue that strain genotyping may open up new avenues for diagnosis and identification of patient subsets.

## Macrophages and the mycobiome in visceral hypersensitivity

Recent publications showed an indispensable role of intestinal CX3CR1^+^ (i.e., fractalkine receptor-expressing) mononuclear phagocytes in providing anti-fungal Th17 immune responses and maintaining intestinal epithelium integrity (Figure 1a).^77,78^ In mice, the latter protection is provided by Cx3cr1^+^ macrophages with high CD11c expression levels. These macrophages insert balloon-like protrusions (BLP) into colonic epithelial cells to sense fungal toxins and metabolites absorbed by these enterocytes. In the presence of fungal toxins, BLP^+^ macrophages instructed epithelial cells to stop fluid absorption, preventing epithelial cell death. Macrophage depletion in distal colon was associated with massive enterocyte apoptosis that was prevented by anti-fungal treatment.^77^ Leonardi *et al*. showed that ablation of intestinal CD11c^+^/Cx3cr1^+^ cells led to mycobiota changes and enhanced susceptibility to dextran sodium sulfate (DSS) colitis that was rescued by fungicide treatment.^78^ In relation to IBS not much is known about a possible role for CD11c^+^/CX3CR1^+^ cells. Microarray analysis of IBS and healthy volunteer sigmoid biopsies indicated elevated expression of CX3CR1 in IBS. But, interpretation of these results is difficult because they were not cell specific.^79^ In addition, if and how overexpression associated with mycobiota dysbiosis is not known. Results obtained in a *Trichinella spiralis* PI-IBS model seem to contradict the above findings on epithelial integrity.^80^ Transfer of post-infectious CD11c^+^ lamina propria mononuclear cells caused mucosal barrier dysfunction and visceral hypersensitivity in naïve recipient mice. However, donor cells were isolated from small intestine. Although, transepithelial dendrites that sample the lumen were indeed reported in small intestine, BLPs observed in colonic CD11c^+^/Cx3cr1^+^ cells do not reach the lumen, suggesting that these two cell types serve different functions.^77,81^

Importantly, *Candida* spp. colonization in mice induced an increase in Th17 cell frequency which dramatically decreased upon depletion of colonic Cx3cr1^+^ cells, confirming their role in anti-fungal immune defense.^78^ Only few reports exist on Th17 immune response in IBS. Berg et al. assessed cytokine levels in homogenized rectal biopsies of IBS patients and controls. They observed, amongst increased presence of several other pro-inflammatory cytokines, that IL-17 was significantly increased in IBS samples.^82^ *In situ* immunohistochemical stainings on descending and rectosigmoid biopsies confirmed enhanced presence of Th17 cells in IBS patients as compared to controls.^83^ Interestingly, using TRPV1-Ai32 optogenetic mice and cutaneous light stimulation it could be shown that activation of TRPV1^+^ neurons is sufficient to elicit, via CGRP-release, a Th17 response. Not only was this response sufficient to increase host defense to *C. albicans*, it also provided regional Type-17 inflammation via a nerve-reflex arc.^84^ These observations suggested, that direct sensory afferent activation by fungi not only signals danger by eliciting a pain response,^56^ but also initiates early protective immunity for adjacent areas. Whether these mechanisms also apply to the intestinal environment and visceral afferents could be a topic for future IBS-related investigations.

Whether colonic CD11c^+^/CX3CR1^+^ cells directly interact with mast cells to induce degranulation is not known, but macrophages may contribute in another unexpected fashion. Macrophages themselves could be a source of histamine. Histidine decarboxylase (HDC) is the only enzyme known to catalyze the conversion of histidine to histamine. Some investigators described HDC expression and histamine release in blood monocytes and bone marrow-derived macrophages.^85–88^ Unfortunately, at this point, there are no data on the possible *in situ* presence of *HDC*-expressing macrophages in IBS tissues. *In vitro*, enhanced histamine release due to luminal factors was successfully shown with LPS, but possible yeast derived triggers were not evaluated for these macrophages.^85,86^

## Inter-kingdom interactions in IBS

Although inter-kingdom interactions are a two-way street, most publications seem to focus on bacteria influencing fungal traits such as biofilm formation, yeast-to-hyphae transition and mucosal invasion. On the other hand, the gut mycobiota also plays an important role in maintaining homeostasis of the bacterial microbiome and gut health. For non-IBS focused but recent overviews on the aforementioned cross-kingdom interactions in health and disease we refer to two excellent reviews.^89,90^ Hong *et al*. addressed positive and negative fungal-bacterial correlations in IBS.^13^ Compared to healthy volunteers, fecal samples of IBS-D showed fewer significant correlations (43 *vs*. 25, resp.). In both groups, the highest number of interactions concerned the genus *Candida*. However, in healthy volunteers 9 out of 12 observed *Candida*-bacteria correlations were negative, whereas in IBS-D all 8 correlations were positive. Moreover, not one of these *Candida*-specific correlations overlapped between the healthy volunteer and IBS-D groups. The overall decline in the number of interactions in IBS-D and changes in specificity of *Candida*-interactions may reflect the microbiome’s inability to maintain homeostasis. The functional relevance of these interactions should be addressed in future investigations, perhaps by using intestinal organoid cultures and microbiota grafting in IBS-like animal models. Possibly, the opposite *Candida* correlations reported by Hong *et al*. can be explained by the earlier reports on *C. albicans* genetic and phenotypic diversity.^14,15^ Thus, we feel that *C. albicans* strain differences should be taken into account when addressing the nature and relevance of altered inter-kingdom interactions.

## Mycobiome-targeted therapy

Direct targeting of the gut fungal community in order to relieve abdominal complaints has, to our knowledge, not yet been tested for patients with IBS. In the IBS-like rodent model, we have shown that classical antifungal treatment does indeed reduce visceral hypersensitivity.^9^ But, considering the increasing fungal resistance to common antifungal medications, other methods should be focused on as well. Administration of the antiparasitic drug miltefosine reduced and prevented visceral hypersensitivity in the same animal model through inference with the gut bacterial and fungal microbial composition, and by reducing mast cell activation.^10^ Similar observations were made for the application of herbal oil mixtures. A preparation of caraway and peppermint essential oils (Menthacarin®) modulated the mycobiome and reduced visceral hypersensitivity in maternal separated rats.^11^ Whether there was a causal relation between modulation of the mycobiota and reduction of complaints remains to be established. In human, the mixtures effect on the gut mycobiome was not yet studied and the same holds true for the herbal mixture STW-5 (Iberogast®) that is indicated for the treatment of IBS and also contains caraway and peppermint.^91^ Furthermore, a meta-analysis on peppermint oil studies in IBS suggested that peppermint oil was more efficacious than placebo for global IBS symptoms and abdominal pain, but mycobiome alterations were never addressed.^92^ Large randomized controlled trials with accompanying mycobiota analysis could provide evidence as to whether herbal-mediated mycobiota modulation is feasible and relevant for patients with IBS.

Dietary restriction of FODMAPs is now widely used in the management of IBS.^6^ Two important mechanisms seem to be involved in the successful application of the low FODMAP diet. In small intestine, FODMAPs are thought to induce enhanced water uptake due to osmotic effects. In colon, microbial fermentation of FODMAPs leads to gas production. Both mechanisms give rise to luminal distension and symptoms in those with visceral hypersensitivity.^93,94^ Despite fungi making up an approximate 1–2% of the luminal biomass,^95^ and although yeast are the prototypical fermentation workhorses in human history, intestinal FODMAP fermentation is always discussed in the context of bacteria only. Especially when knowing that high *Candida* spp. abundance strongly associated with recent consumption of carbohydrates,^96^ investigations addressing the relative involvement of yeast in the success of the low FODMAP diet are eagerly awaited. Alternatively, and based on the aforementioned inter-kingdom interactions, bacterial probiotics may be used to interfere with intestinal yeasts.^90^ In a recent systemic meta-analysis, McFarland *et al*. aimed to determine, when taking into account the recommendations from the European Society for Pediatric Gastroenterology, Hepathology and Nutrition (ESPGHAN) and the American Gastroenterology Association (AGA), which probiotics are safe and effective for the treatment of IBS. Only randomized, controlled trials (RCTs) with at least one strain-specific confirmatory RCT and common IBS outcome measures such as abdominal pain scores were included.^97^ Only 3 single strain bacterial probiotics showed significant changes in abdominal pain scores and/or frequency of abdominal pain relief: *Lactobacilli plantarum* 299 v, *Lactobacillus rhamnosus* GG (LGG) and *Bacillus coagulans* MTCC5260. Although their working mechanisms in IBS are largely unknown, for two of these strains (*L. plantarum* 299 v and LGG) *in vitro* experiments have shown effects on *Candida* growth, morphogenesis, and adhesion, and these outcomes may also be relevant to IBS.^98–101^ We suggest, that future selection procedures of probiotics for IBS clinical trials should include *in vitro* yeast-related read outs. Importantly, it was shown that the efficacy of probiotics is both strain- and disease-specific.^102^ Thus, multiple strains of the same species should be included in *in vitro* experiments and selected strains should be tested in preclinical models prior to clinical evaluation. In addition to probiotic bacteria, some yeasts are suggested to have probiotic effects as well. At present, only a very limited set of yeast species have a qualified presumption of safety (QPS) by the European Food Safety Agency (EFSA) for viable usage. These include *Candida kefyr* (also known as *Kluyveromyces marxianus*) and *Saccharomyces cerevisiae* (including *S. boulardii* CNCM I-7454 and *S. cerevisiae* CNCM I-3856).^103,104^ In the McFarland meta-analysis on probiotic RCTs in IBS, the latter two *S. cerevisiae* strains showed significant efficacy for abdominal pain management in ≥ 2 RCTs.^97^ Although the responsible mechanisms remain elusive, both strains are known to affect *C. albicans* as well as *albicans*-related host immune responses.^104,105^ We suggest that the efficacy of yeast probiotics in IBS may be further enhanced when probiotic strain selection is performed with *C. albicans* or other IBS-suspect fungal species in mind. Relevant aspects such as *C. albicans* adhesion to intestinal epithelial cells, yeast-to-hypha transition and host immune responses can be targeted during such probiotic evaluations.

## Perspective

Although still limited in number, recent publications indicated intestinal mycobiota alterations in IBS. Similar findings in IBD initiated a promising new field of mycobiome-oriented research, and the same may happen in IBS. Investigations performed in an IBS-like rat model already suggested that the observed mycobiota changes may be relevant for abdominal pain and addressed possible mechanisms and therapeutic approaches. In IBS there is a relative increase of intestinal *C. albicans* and genotyping of fecal *C. albicans* strains indicated clustering of IBS-derived strains as compared to healthy volunteers. Traditionally, there is a lot of attention for *C. albicans* in the broader mycobiome field of investigations. Recent insights on *C. albicans*/macrophage and *C. albicans*/epithelial cell interactions as well as *C. albicans-*mediated afferent-activation should be addressed in relation to IBS. Taken together, we suggest that fungal feelings in IBS should no longer be ignored, but investigated instead.
